# Myofibroma of the Gingiva: A Rare Case Report and Literature Review

**DOI:** 10.1155/2015/243894

**Published:** 2015-03-30

**Authors:** Vaishali Narayen, Syed Afroz Ahmed, Charu Suri, Shahela Tanveer

**Affiliations:** Department of Oral and Maxillofacial Pathology, Sri Sai College of Dental Surgery, Vikarabad 501101, India

## Abstract

Myofibromas are benign uncommon fibroblastic tumors of the soft tissue, bone, or internal organs affecting all ages. These lesions histopathologically may mimic many other soft tissue tumors of the oral cavity such as spindle cell tumors of neurogenic and smooth muscle cell origin, thus leading to misdiagnosis and mistreatment. This case report describes a rare benign tumor, which presented as a soft tissue swelling on posterior gingiva. Surgical excision of the lesion was carried out under local anaesthesia. Histopathologic and immunohistochemical examination confirmed the diagnosis of myofibroma. Myofibroma should be included in the clinical differential diagnosis of masses of the oral soft tissues; however immunohistochemical examination is essential to establish an accurate diagnosis.

## 1. Introduction

Myofibroma and myofibromatosis are benign fibroblastic and myofibroblastic tumors that may develop in the soft tissue, bone, or internal organs affecting all ages. In 1989, a solitary case of this tumor was delineated by Smith et al. and was termed “myofibroma” [1]. Afterwards, the terms “myofibromatosis” and “myofibroma” were adopted by the World Health Organization. Myofibroma involves predominantly the head and neck region (36%) or the trunk; however cases affecting the oral cavity are rare and present with a wide differential diagnosis. The purpose of this paper is to report a case of myofibroma occurring in the left mandibular posterior region and to describe its clinical, histopathologic, and immunohistochemical features.

## 2. Case Presentation

**Figure 1:**
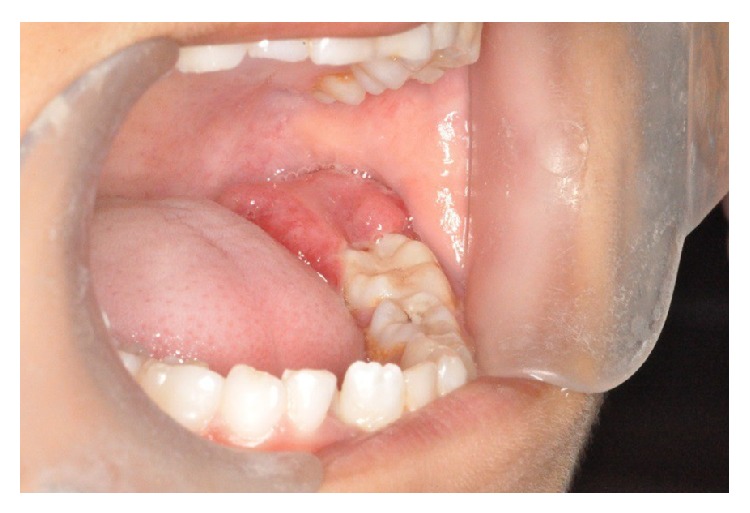
Intraoral picture showing growth in the lower left posterior region on lingual and distal aspect of 36.

**Figure 2:**
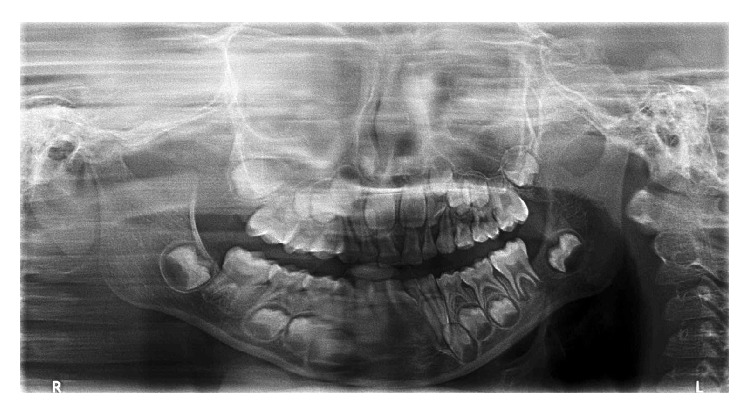
Orthopantomograph showing no bony involvement.

A 7-year-old girl reported to our hospital with a complaint of pain and swelling in the mandibular left posterior region for one month. The patient gave a history of bleeding from the swelling, which was aggravated upon brushing and chewing. The past medical and dental history were noncontributory. Clinical examination revealed a solitary, well defined, soft tissue growth on the posterior gingiva measuring about 2.5 × 1.5 cm, extending from the lingual and distal aspect of 36 to posteriorly 1 cm short of the anterior border of the ramus. The growth was sessile, dark red in colour, firm in consistency, tender on palpation, and nonfluctuant (Figure 1). Based on the history and clinical presentation a provisional diagnosis of pyogenic granuloma was made. The orthopantomograph showed no bony involvement (Figure 2). The initial clinical differential diagnosis included traumatic fibroma, peripheral giant cell lesion, neurofibroma, schwannoma, and hemangiopericytoma. Excisional biopsy was performed under local anaesthesia.

**Figure 3:**
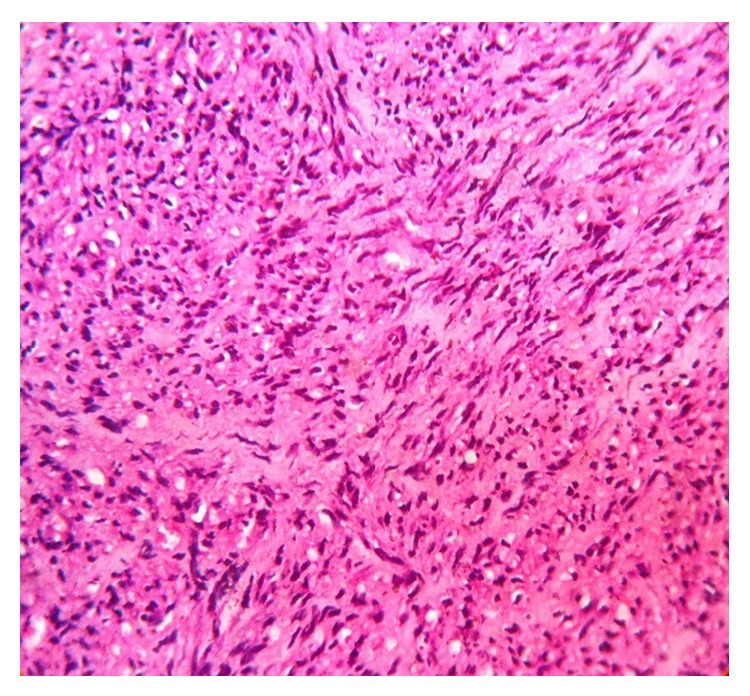
Intertwining bundles of spindle cells with abundant extracellular collagenous matrix (H&E stain, original magnification ×40).

**Figure 4:**
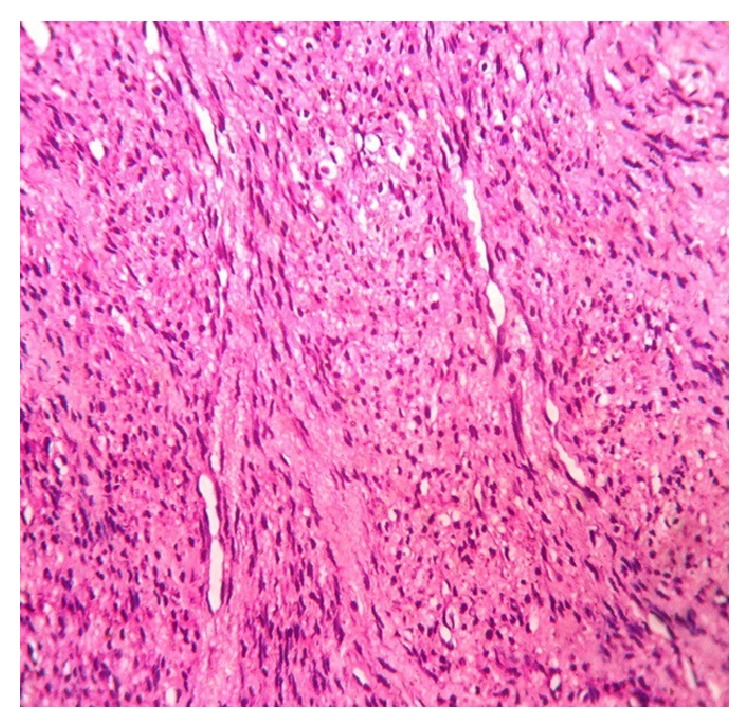
Groups of densely packed cells adjacent to angular hemangiopericytoma-like blood vessels (H&E stain, original magnification ×40).

Histopathological examination revealed anastomosing squamous surface epithelium with elongated rete ridges. The tumor was composed mainly of streaming and interlacing fascicles of spindle-shaped cells resembling fibroblasts or smooth muscle cells, suggesting myofibroblastic differentiations (Figure 3), and a few mitotic figures. In some areas, the lesional cells appeared to be round or polygonal and closely packed (Figure 4). The findings were suggestive of myofibroma but a definitive diagnosis was warranted. Additional serial sections were obtained for immunohistochemical studies.

**Figure 5:**
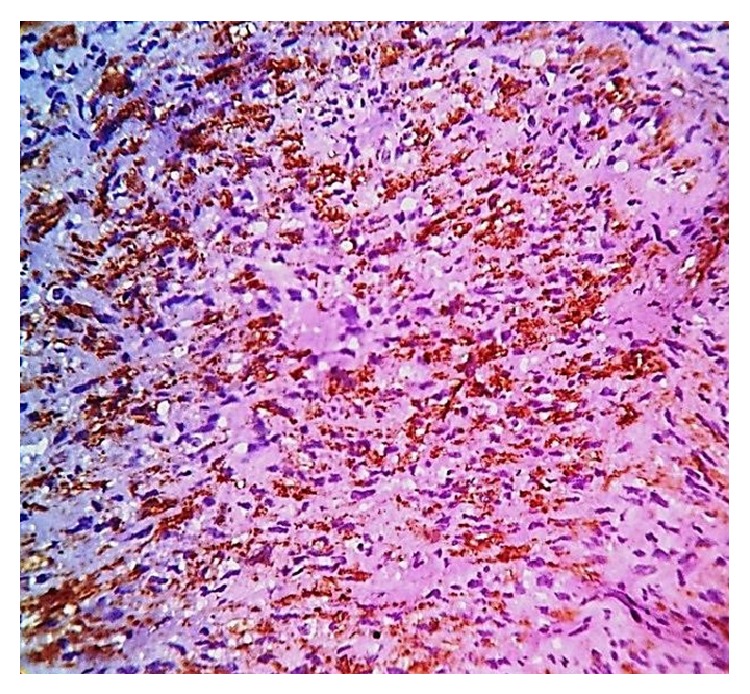
Bundles of lesional spindle cells intensely positive for alpha-smooth muscle actin (original magnification ×40).

**Figure 6:**
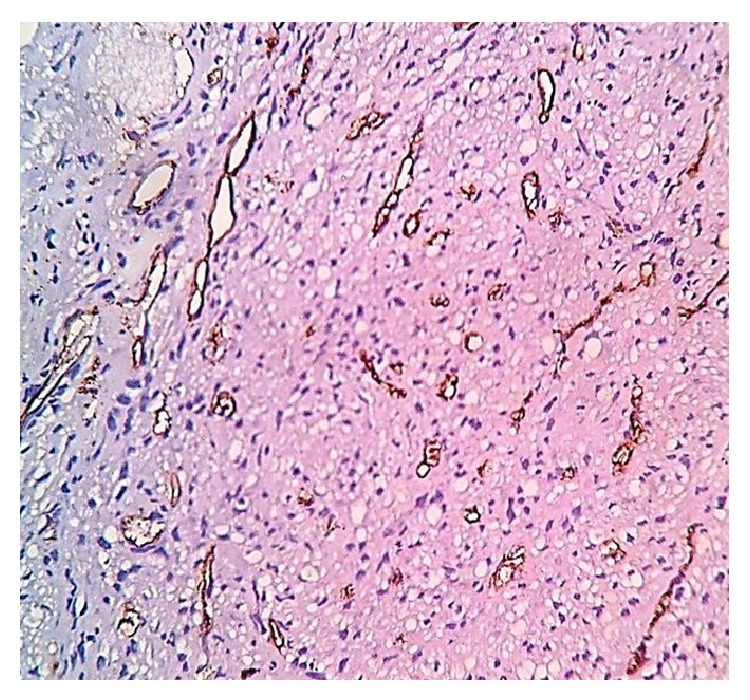
Lesional cells are negative for CD34. Positive stain highlights the extensive vascular network of the lesion (original magnification ×40).

Immunohistochemistry showed a positive reaction in the tumor cells for *α*-smooth muscle actin (*α*-SMA) (Figure 5) but no immunoreactivity for antibodies directed against CD34 (Figure 6). We would have also done IHC for smooth muscle-specific myosin, in order to rule out leiomyoma, which would also be SMA-positive. It is still likely that the lesion is myofibroma, however. Based on the histopathologic and immunohistochemical features, a diagnosis of myofibroma involving the left gingiva was made.

## 3. Discussion

The term myofibromatosis was first used by Daimaru et al. in the year 1989, and later, in the same year, a solitary case of this tumor was delineated by Smith et al. who termed it as myofibroma [2]. Myofibroma/myofibromatosis are benign fibroblastic tumors composed of myeloid cells with thin blood vessels involving the soft tissues, bones, and internal organs. The solitary proliferation is termed as myofibromas and its multicentric counterpart is called myofibromatosis [1]. The present case describes a solitary lesion of myofibroma in a 7-year-old female patient. Myofibroma is the most common fibrous proliferation in childhood with a predisposition for the soft tissues of the head and neck region [3]. Myofibroma may appear from new born to old age [4], although 90% of these lesions appear before 2 years of age. About 17% were reported during the first year of life. Given that most of the lesions were reported during the first two decades (55%), the entity of myofibroma of the oral soft tissues is a tumor of children and teenagers rather than infants.

Generally, myofibromas are more common in males than in females [4, 5] with the male : female ratio being 2 : 1 [3] and the case reported here is that of female patient. The tongue is most commonly involved (30%) followed by buccal mucosa (20%) and is rarely seen on the gingiva as in this case [3]. Only few cases of this tumor are reported to occur in gingiva [6]. In a recent study done by Aiki et al., it was found that, among 94 cases of review, myofibromas involved the mandible (33%), gingiva (23%), tongue (15%), cheek or buccal mucosa (12%), palate (8%), lip (4%), and other areas (5%) in that order [7].

According to Vered et al., the histopathologic features of myofibroma of the oral soft tissues are in accordance with those described for myofibroma from other parts of the body. However, one major difference was that while necrosis and calcifications were a common finding in tumors involving other anatomic sites, only four cases of myofibroma of the oral soft tissues demonstrated necrosis and/or calcifications [8]. Areas of necrosis are suggested to be associated with spontaneous regression of myofibroma [9]. In oral myofibroma, only one lesion with necrosis and calcifications has been reported to regress spontaneously after partial excision. The occurrence of spontaneous regression of myofibromas may be mediated by apoptosis [9], which is seen in many processes of normal embryonic and postnatal normal development. This may explain why myofibromas that are present at birth or appear shortly after tend to regress spontaneously. It can be assumed that myofibroblasts that are influenced by various temporal and spatial factors become more resistant to apoptosis as the patient ages. Therefore, lesions of myofibroma of the oral soft tissues that generally develop in children and older patients do not tend to regress spontaneously.

Histopathologically myofibroma exhibits a biphasic pattern of light and dark-stained areas. The light area mainly consists of spindle cells with eosinophilic cytoplasm and tapering or cigar-shaped nuclei, arranged in short fascicles or whorls and nodules, at the periphery of the lesion. However, sometimes these cells are distributed haphazardly throughout the lesion. In contrast, the more intensely stained areas, located more centrally, consist of round cells or small spindle cells arranged around thin-walled, irregularly branching, hemangiopericytoma-like blood vessels. These cells have basophilic nuclei, small eosinophilic cytoplasm, and indistinct cell margins [3]. In some cases the light and dark areas are not separate and the two cell subpopulations are intermixed [8]. Mitotic figures are only rarely observed but lesions deeply located are often ill-defined and focally tend to infiltrate the surrounded tissue [10]. Immunohistochemically, myofibroma cells express *α*-SMA, muscle-specific actin, and vimentin and are negative for desmin, S-100 protein, and CD34 [3, 8, 10, 11]. In our case, histopathologic, histochemical, and immunohistochemical findings fulfil the criteria for a diagnosis of myofibroma of oral soft tissues.

The histopathologic differential diagnosis of myofibroma of the oral soft tissues includes leiomyoma, schwannoma, nodular fasciitis, benign fibrous histiocytoma, solitary fibrous tumor, desmoid type of fibromatosis, and infantile fibrosarcoma [3, 8]. The neoplastic cells of vascular leiomyoma are positive for desmin in contrast to negative myofibroma. Furthermore, Masson's trichrome stain in vascular leiomyoma shows the presence of delicate fibrous tissue that surrounds smooth muscle cells and in the septa between masses of neoplastic cells. This distribution is different from that of the thick fibrous bundles, with random, irregularly intersecting angles prominently observed in myofibroma [11]. Schwannoma and neurofibroma do not contain hemangiopericytoma-like blood vessels and the neoplastic cells are positive for S-100 protein. Nodular fasciitis demonstrates extravasated red blood cells, a myxoid stroma, and chronic inflammatory cells, features that are absent in myofibroma [8]. Benign fibrous histiocytoma consists of fibroblast-like spindle cells and histiocytic-like cells arranged in a storiform pattern. These cells express factor XIIIa and *α*1-antitrypsin [3]. Solitary fibrous tumor may be differentiated from myofibroma because its neoplastic cells express CD34 [12]. Unlike myofibroma, desmoid-type fibromatosis is not characterized by biphasic cellular populations. Finally, infantile fibrosarcoma is characterized by the presence of uniform spindle cells that form fascicles, admixed with areas of focal necrosis and hemorrhage, features that are not normally seen in myofibroma [3].

Treatment for the typical solitary lesion is excisional biopsy or simple excision. Local recurrence has been reported in 7% to 31% of excised cases of myofibromatosis [13]; however those recurrences are thought to be mostly caused by multicentric tumor feature or insufficient excision. Chemotherapy or radiation has been seldom used for myofibromatosis except for a few cases with recurrence or nonresectable lesion [14].

The prognosis of this tumor is typically excellent for solitary myofibromas. On the other hand, the cases with multicentric visceral lesions, as myofibromatosis, may sometimes show an aggressive and sometimes fatal outcome.

## 4. Conclusion

Since the prognosis of this tumor is variable and can range from excellent to sometimes fatal (not for solitary myofibroma), an early and correct diagnosis is mandatory. In the present case, the lesion was found on the posterior gingiva, lingual and distal to 36, a site common to many different types of tumors, thereby resulting in a wide range of differential diagnostic possibilities. The histopathologic differential diagnosis consists of spindle cell tumors of neurogenic and smooth muscle cell differentiation, as well as other myofibroblastic lesions like myofibroblastoma, intranodal myofibroblastoma, angiomyofibroblastoma, and dermatomyofibroma with variable biologic behaviour ranging from reactive to benign-aggressive and malignant tumors, making immunohistochemistry the mainstay for diagnosis of such tumors.
